# Genome Sequences of *Serratia* Strains Revealed Common Genes in Both Serratomolides Gene Clusters

**DOI:** 10.3390/biology9120482

**Published:** 2020-12-20

**Authors:** Catarina Marques-Pereira, Diogo Neves Proença, Paula V. Morais

**Affiliations:** Department of Life Sciences, Centre for Mechanical Engineering, Materials and Processes, University of Coimbra, Calçada Martim de Freitas, 3000-456 Coimbra, Portugal; catarina.103@gmail.com (C.M.-P.); pvmorais@uc.pt (P.V.M.)

**Keywords:** *Serratia*, genome, serrawettin, biosynthetic gene cluster, serratomolides, *swrW* gene, *swrA* gene

## Abstract

**Simple Summary:**

Biosurfactants are amphiphilic molecules produced by microorganisms with a hydrophilic and a hydrophobic group, able to reduce surface tension. These molecules are largely used in the environmental, food, pharmaceutical, medical, and cleaning industries, among others. *Serratia* strains are ubiquitous microorganisms with the ability to produce biosurfactants, such as serrawettins. These extracellular lipopeptides are described as biocides against many bacteria and fungi. This work used comparative genomics to determine the distribution and organization of the serrawettins W1 and W2 biosynthetic gene clusters in all the 84 publicly available genomes of the *Serratia* genus. Here, the serrawettin W1 gene clusters’ organization is reported for the first time. The serrawettin W1 biosynthetic gene *swrW* and serrawettin W2 biosynthetic gene *swrA* were present in 17 and 11 *Serratia* genomes, respectively. The same genes in the biosynthetic clusters frame the *swrW* and *swrA* biosynthetic genes. This work identified four genes common to all serrawettin gene clusters, highlighting their key potential in the serrawettins biosynthetic process.

**Abstract:**

*Serratia* strains are ubiquitous microorganisms with the ability to produce serratomolides, such as serrawettins. These extracellular lipopeptides are described as biocides against many bacteria and fungi and may have a nematicidal activity against phytopathogenic nematodes. Serrawettins W1 and W2 from different strains have different structures that might be correlated with distinct genomic organizations. This work used comparative genomics to determine the distribution and the organization of the serrawettins biosynthetic gene clusters in all the 84 publicly available genomes of the *Serratia* genus. The serrawettin W1 and W2 gene clusters’ organization was established using antiSMASH software and compared with single and short data previously described for YD25^T^ *Serratia*. Here, the serrawettin W1 gene clusters’ organization is reported for the first time. The serrawettin W1 biosynthetic gene *swrW* was present in 17 *Serratia* genomes. Eighty different coding sequence (CDS) were assigned to the W1 gene cluster, 13 being common to all clusters. The serrawettin W2 *swrA* gene was present in 11 *Serratia* genomes. The W2 gene clusters included 68 CDS with 24 present in all the clusters. The genomic analysis showed the *swrA* gene constitutes five modules, four with three domains and one with four domains, while the *swrW* gene constitutes one module with four domains. This work identified four genes common to all serrawettin gene clusters, highlighting their essential potential in the serrawettins biosynthetic process.

## 1. Introduction

Surfactants are amphiphilic molecules with a hydrophilic and a hydrophobic group, able to reduce surface tension. These molecules are largely used in the environmental, food, pharmaceutical, medical, and cleaning industries, among others [1,2,3]. Biosurfactants are secondary metabolites produced by bacteria, yeast, or fungi, capable of reducing the surface tension of extracellular media [4]. They can have a variety of structures, mainly divided into six major groups, namely, glycolipids, lipopolysaccharides, lipopeptides and phospholipids, and hydroxylated and cross-linked fatty acids [5]. When compared with synthetic surfactants, biosurfactants have higher biodegradability, lower toxicity, and higher activity at extreme conditions, such as high temperatures, pH, and salinity [6].

Bacterial species of the genus *Serratia* belonging to the family *Enterobacteriaceae* are Gram-negative, facultative anaerobic, and rod-shaped bacteria [7,8]. They have been isolated from different environments, such as water, soil, plants, insects, and vertebrates [9]. Surfaces in these habitats have a variety of characteristics and can be hydrophobic, hydrophilic, fractal, smooth, and axenic, among others. To colonize them, bacteria from the genus *Serratia* produce biosurfactants (extracellular lipopeptides) as the serratomolides serrawettin W1 [10], serrawettin W2 [4], and serrawettin W3 [11]. Mutants for the serratomolides biosynthetic genes failed to form colonies on nutritive agar plates and these lipopeptides were suggested as promoters of a new type of spreading growth [4].

Both the serrawettin W1 and W2 biosynthetic genes (*swrW* and *swrA* genes) belong to a Non-Ribosomal Peptide Synthetase (NRPS) gene cluster [12,13]. Condensation, adenylation, thiolation, and thioesterase domains are detectable in both serrawettin gene clusters. Along with other proteins, such as PPTase or acyl carrier proteins (ACP) [12,14], serrawettin W1 and W2 biosynthetic proteins are able to synthesize serrawettin W1 and W2 lipopeptides. Serrawettin W1 has a symmetric structure composed of two serine amino acids and two fatty acids (3-hydroxydecanoic) [4] while serrawettin W2 is a cyclic peptide containing five amino acids. This lipopeptide promotes flagellum spreading growth, contributing specifically to surface bacterial translocation [4], and was able to demonstrate antimicrobial activity against many bacteria and fungi and an antitumoral activity against Hela cells [13]. The *Serratia* strains producing serrawettin W1 showed the highest broad-spectrum antimicrobial activity against clinical, food, and environmental bacterial pathogens compared with the *Serratia* strains producing serrawettin W2 [15]. A strain of *S. marcescens* showed the capacity to inhibit an endophytic fungus due to its production of compounds, namely, serrawettins [16].

Due to the limited information on this topic, in this study, we performed a comparative genomic analysis to determine the distribution and the organization of the serrawettins biosynthetic gene clusters in all the 84 publicly available genomes of the *Serratia* genus. Here, we report a deep analysis of the serrawettin W1 and W2 gene clusters and establish their organization using antiSMASH software. In particular, for the first time, we show the serrawettin W1 gene clusters’ organization. These analyses showed the presence of four genes common to all the serrawettin gene clusters, highlighting their key potential in the serrawettins biosynthetic process.

## 2. Materials and Methods

### 2.1. Bacterial Strains, Data Collection, and Genomes

Eighty-four *Serratia* genomes available on NCBI, representing all the publicly available genomes, were included in this study (Table S1), obtained from 49 different hosts and isolated from 29 countries [13,17,18,19,20,21,22,23,24,25,26,27,28,29,30,31,32,33,34,35,36,37,38,39,40,41,42,43,44,45,46,47,48,49].

Serrawettin W1 and W2 biosynthetic gene cluster prediction was performed in all the 84 complete and draft *Serratia* genomes publicly available, using the web platform antiSMASH 3.0 [50]. This input dataset includes strains belonging to *S. marcescens*, *S. liquefaciens*, *S. grimesii*, *S. nematodiphila*, *S. plymuthica*, and *S. ureilytica*, as well as strains not characterized to the species level. AntiSMASH analysis output was examined to identify the serrawettins biosynthetic genes clusters. Therefore, to find the *swrW* and *swrA* genes, BLAST analysis was performed for all protein sequences, codified by each gene of the NRPS predicting metabolite clusters, using the NCBI [51] database. Cluster boundaries were predicted with the CASSIS algorithm specified for the NRPS domains [50].

### 2.2. Phylogenetic Analysis

The 16S rRNA sequences of the 47 *Serratia* strains, which showed the presence of serrawettins genes in their genomes (Table 1), were selected for phylogenetic analysis. These sequences were compared with the sequences available in the EMBL/GenBank database using the BLASTN network services [51], and with sequences available at the Eztaxon-e server (http://eztaxon-e.ezbiocloud.net/) [52]. Sequences were aligned within the SINA alignment service [53]. Sequences were included in the 16S rRNA-based Living Tree Project (LTP, release 115) database (http://www.arb-silva.de/projects/living-tree/) by parsimony implemented in the ARB software package version 5.5 [54]. Phylogenetic dendrograms of this study strains and closest reference sequences were constructed using the Neighbor-Joining and Randomized Axelerated Maximum Likelihood (RAxML) method with the GTRGAMMA model [55] included in the ARB software [54].

### 2.3. Serrawettins Biosynthetic Gene Clusters Analysis

The NRPS gene cluster, where both the serrawettin W1 and W2 biosynthetic genes belong, was analyzed here by using antiSMASH software [50]. The reconstruction of the domains, modules, and structures of the serrawettins was performed by using Phyre2 tridimensional prediction [56], using the serrawettin W1 biosynthetic protein from *Serratia* sp. AS13, and W2 from *Serratia* sp. PWN146. PubChem 2D was used to explore the chemical information of the serrawettins using the bacterial models mentioned above.

The concatenated amino acid sequences of each serrawettin biosynthetic gene cluster were organized by protein identification in the same order to allow a cluster alignment with ClustalW in MEGAX software [57]. The serrawettin W1 biosynthetic proteins from 17 *Serratia* genomes and serrawettin W2 biosynthetic proteins from 11 *Serratia* genomes were separately aligned. The evolutionary relationship between the clusters and between the serrawettin biosynthetic protein sequences were established using the Neighbor-Joining method, Poisson model [58], in MEGAX software [57]. The aligned and organized cluster genes were represented in an evolutionary tree according to the size, direction, and accession numbers identified with the NCBI BLAST database [51]. Functions of the core proteins in all clusters were searched on UniProt [59].

## 3. Results

### 3.1. Bacterial Phylogeny and Comparative Genomics of Serratia spp.

**Figure 1:**
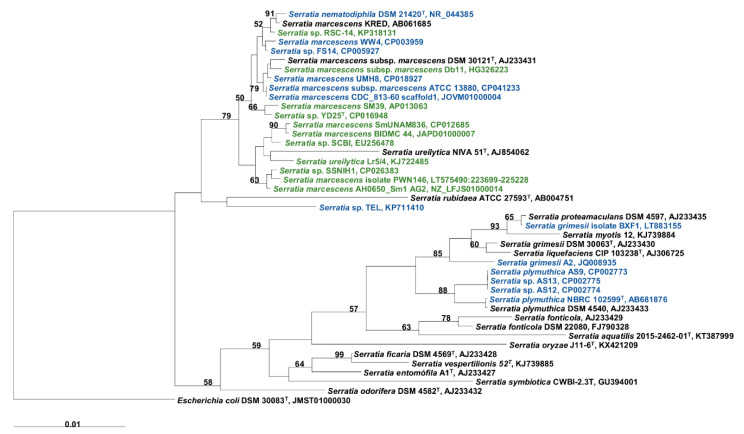
A phylogenetic dendrogram based on a comparison of the 16S rRNA gene sequence of the *Serratia* strains used in this study and the type of strains. The tree was created using the Neighbor-Joining method in ARB software. The numbers on the tree indicate the percentages of bootstrap sampling, derived from 1000 replications; values below 50% are not shown. In blue are shown the *Serratia* strains that showed the presence of the serrawettin W1 biosynthetic gene cluster and in green are shown the *Serratia* strains that showed the presence of the serrawettin W2 biosynthetic gene cluster. The type species *Escherichia coli* DSM 30083^T^ was used as the outgroup. Scale bar, 1 inferred nucleotide substitution per 100 nucleotides.

All the 84 *Serratia* strains selected for this study had their genome publicly available at NCBI. According to the Neighbor-Joining and Maximum-Likelihood phylogenetic trees based on 16S rRNA gene sequences, 14 strains belong to *S. marcescens*, two strains belong to *S. plymuthica*, two strains belong to *S. grimesii*, one strain belongs to *S. liquefaciens*, one stain belongs to *S. nematodiphila*, one strain belongs to *S. ureilytica*, and 63 strains could not be assigned to species level due to a similarity lower than 97% (Figure 1). Genomes of the *Serratia* strains have a size from 5.0 Mbp to 7.7 Mbp and the G + C content varies from 45.9 to 60.1 mol%.

Bioinformatic analysis through antiSMASH software showed that the serrawettin W1 biosynthetic gene was present in 17 *Serratia* genomes, strains ATCC 13880, CDC_813-60 DP21, UMH8, IOMTU 115, DSM 21420, VGH107, EGD-HP20, WW4, FS14, BIDMC81, TEL NODE_13, NBRC 102599^T^, BXF1, A2, AS13, AS9, and AS12 (Table 1 and Table S2). The serrawettin W2 biosynthetic gene was present in 11 *Serratia* strains, PWN146, SSNIH1, SM39, SmUNAM836, BIDMC 44, Lr5/4 LG59, RSC-14, AH0650_Sm1 AG2, Db11, SCBI, and YD25^T^ (Table 1 and Table S3).

### 3.2. Serrawettin W1 Biosynthetic Gene Clusters

**Figure 2:**
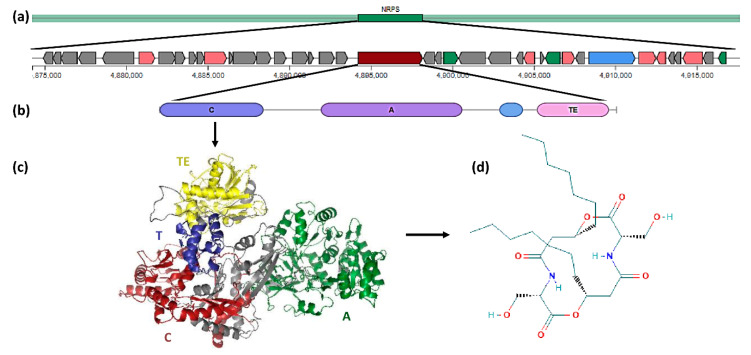
Serrawettin W1: from the biosynthetic gene cluster to the structure of serrawettin W1. Genetic organization of the genome of Serratia strain AS13 by antiSMASH analysis, Phyre2 tridimensional prediction of serrawettin W1 biosynthetic protein, and PubChem 2D structure of serrawettin W1. (**a**) Serrawettin W1 biosynthetic gene cluster with identification of the srwW core gene; (**b**) *swrW* gene organization: C, condensation domain; A, adenylation domain; T, thiolation domain; and TE, thioesterase domain; (**c**) biosynthetic protein with the condensation, adenylation, thiolation, and thioesterase domains; (**d**) serrawettin W1 2D structure.

All serrawettin W1 biosynthetic gene clusters were identified as NRPS clusters. Moreover, the bioinformatic analysis predicted an architecture including the domains condensation (C), adenylation (A), thiolation (T), and thioesterase (TE) in all the serrawettin W1 biosynthetic genes (Figure 2).

To confirm the identification of the *swrW* gene revealed by antiSMASH, each *swrW* was queried to NCBI BLASTP, in order to find the closest relative and determine the identity percentage (Table 2 and Table S2). The protein from the serrawettin W1 biosynthetic gene (*swrW*) showed an identity percentage that ranges from 77.79% to 100% as the closest identification by using BLASTP (Table 2).

**Figure 3:**
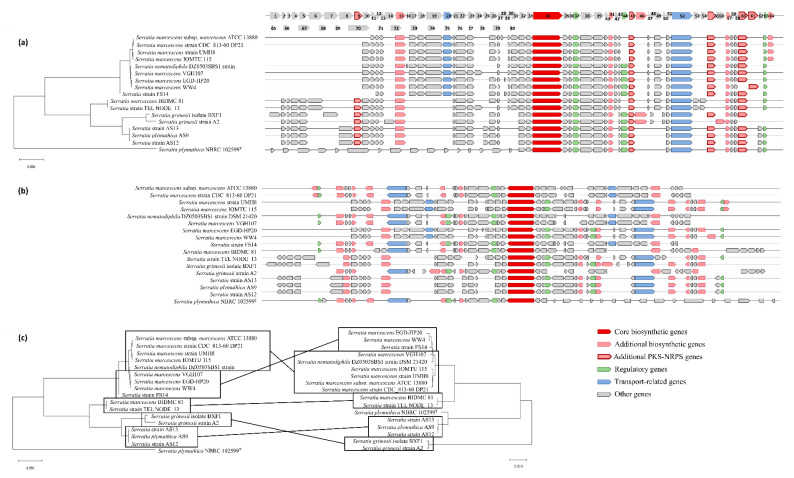
Serrawettin W1 gene cluster analysis. The phylogenetic relationship was established with Mega X software by the Neighbor-Joining method on aligned serrawettin W1 gene clusters of *Serratia* strains ATCC 13880, CDC_813-60 DP21, UMH8, IOMTU 115, DSM 21420, VGH107, EGD-HP20, WW4, FS14, BIDMC81, TEL NODE_13, NBRC 102599, BXF1, A2, AS13, AS9, and AS12. (**a**) Phylogenetic tree based on protein sequences of the serrawettin W1 biosynthetic gene cluster on an established genetic organization. (**b**) Serrawettin W1 biosynthetic gene clusters based on natural genetic organization. (**c**) Comparison of the phylogenetic tree based on protein sequences of the serrawettin W1 biosynthetic gene clusters (left) with the phylogenetic tree of the serrawettin W1 biosynthesis protein (right). The scale bar of 0.050 infers the nucleotide substitutions per 100 nucleotides.

Eighty different genes from the serrawettin W1 biosynthetic gene cluster were identified. Fifteen genes are common to all 17 gene clusters (Figure 3 and Table S4), such as genes encoding for murein hydrolase effector protein LrgB and murein hydrolase regulator LrgA, both with hydrolase activity; LysR regulatory protein with DNA-binding transcription factor activity; a sodium-hydrogen antiporter and xanthine-uracil-vitamin C permease, both with transmembrane transport activity; glyoxalase–bleomycin resistance protein and glutathione S-transferase domain protein, both with a dioxygenase activity; 3-oxoacyl-(acyl-carrier-protein) reductase; single-stranded DNA-binding protein; exonuclease ABC subunit A; maltose O-acetyltransferase; and aromatic amino acid aminotransferase. Not considering strain NRBC 102599^T^, an additional seven genes were found to be common to all strains in the serrawettin W1 biosynthetic gene cluster (Figure 3). Moreover, 15 genes are exclusive to the *S. plymuthica* NBRC 102599^T^ biosynthetic gene cluster (Figure 3).

The relationship between strains established based on the analysis of the concatenated genes of the W1 biosynthetic gene cluster defined the same clusters as the relationships defined based on the *swrW* gene analysis, except for *S. plymuthica* NBRC 102599^T^, which is discordant. The position of *S. plymuthica* NBRC 102599^T^ in W1 phylogenetic tree highlights the different gene composition of the W1 biosynthetic cluster of the strain. On the other hand, in the *swrW* phylogenetic tree, *S. plymuthica* NBRC 102599^T^ forms a sister group with *Serratia* strains AS13, AS9, and AS12 (Figure 3).

In the serrawettin W1 biosynthetic gene cluster, software prediction identified four genes involved in PKS-NRPS (PolyKetide Synthases Non-Ribosomal Peptide Synthetases), encoding for enoylreductase quinone oxidoreductase (only present in seven strains), ketoreductase 3-oxoacyl-(acyl-carrier-protein) reductase (present in all strains), enoylreductase dehydrogenase (absent in two strains), and aromatic amino acid aminotransferase (present in all strains) (Table 3 and Table S4, Figure 3).

### 3.3. Serrawettin W2 Biosynthetic Gene Clusters

**Figure 4:**
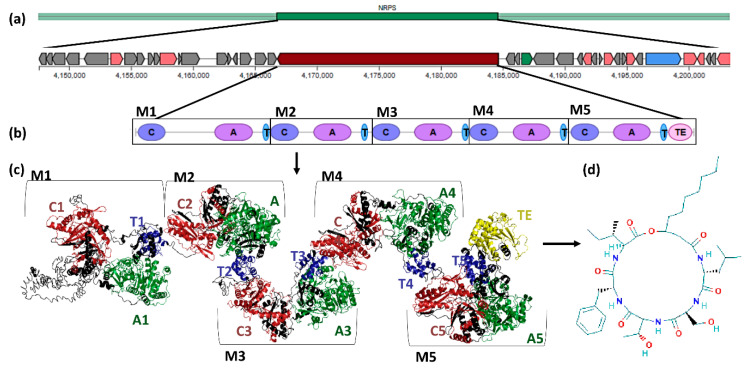
Serrawettin W2: from the biosynthetic gene cluster to the structure of serrawettin W2. Genetic organization of the genome of *Serratia* strain PWN146 by antiSMASH analysis, Phyre2 tridimensional prediction of the serrawettin W2 biosynthetic protein, and PubChem 2D structure of serrawettin W2. (**a**) Serrawettin W2 biosynthetic gene cluster with identification of the *swrA* core gene; (**b**) *swrA* gene organization: five modules (M1–M5) with C, condensation domain; A, adenylation domain; T, thiolation domain; and TE, thioesterase domain; (**c**) biosynthetic protein with five modules (M1–M5), each composed of condensation, adenylation, and thiolation domains, and in the last module an additional thioesterase domain.

Every serrawettin W2 biosynthetic gene (*swrA*) showed an architecture composed of five modules, each with a condensation (C1, C2, C3, C4, and C5), adenylation (A1, A2, A3, A4, and A5), and thiolation (T1, T2, T3, T4, and T5) domain. Module 5 has an additional thioesterase (TE) domain. This organization is shared by all the serrawettin W2 biosynthetic genes (Figure 4).

To confirm the identification of the *swrA* gene revealed by antiSMASH, each *swrA* was, as mentioned above, queried to NCBI BLASTX, in order to find the closest relative and determine the identity percentage (Table 4 and Table S3). The protein coded by the biosynthetic genes of serrawettin W2 (*swrA*) showed an identity percentage that ranges from 76.38% to 99.4% as the closest identification by using BLASTX (Table 4).

**Figure 5:**
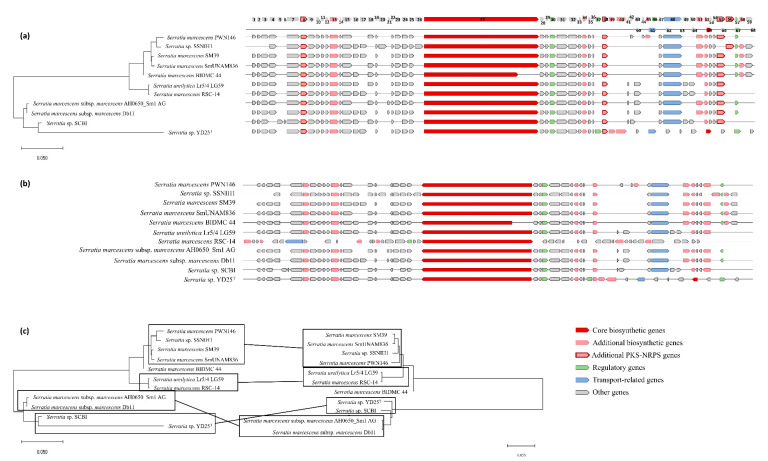
Serrawettin W2 gene cluster analysis. The phylogenetic relationship was established with Mega X software by the Neighbor-Joining method on aligned serrawettin W1 gene clusters of *Serratia* strains PWN146, SSNIH1, SM39, SmUNAm836, BIDMC44, Lr5/4 LG59, RSC-14, AH0650, Db11, SCBI, and YD25^T^. (**a**) Phylogenetic tree based on protein sequences of serrawettin W2 biosynthetic gene clusters on an established genetic organization. (**b**) Serrawettin W2 biosynthetic gene clusters based on natural genetic organization. (**c**) Comparison of the phylogenetic tree based on protein sequences of the serrawettin W1 biosynthetic gene clusters (left) with the phylogenetic tree of the serrawettin W1 biosynthesis protein (right). The scale bar of 0.050 infers nucleotide substitutions per 100 nucleotides.

Sixty-eight genes were identified in the serrawettin W2 biosynthetic gene clusters (Figure 5 and Table S5). Twenty-four genes were present in all the strains’ gene clusters (Figure 5), namely, the genes encoding for RNase E specificity factor CsrD, with aminotransferase activity; acrylyl-CoA reductase; sulfoxide reductase subunit YedY and YedZ; 3-dehydroquinate dehydratase, involved on aromatic amino acids biosynthesis; biotin carboxyl carrier protein and carboxylase subunit, both components of the acetyl-CoA carboxylase complex; sodium/pantothenate symporter; DNA-binding proteins; exported proteins involved in cell adhesion; lipid A biosynthesis lauryl acyltransferase, with catalytic activity; glutamine amidotransferase; murein effectors LrgA and LrgB, with hydrolase activity; and LysR transcriptional regulator, with carbonate dehydratase.

The phylogenetic relationship between the strains was established based on the analysis of the concatenated genes of the W2 biosynthetic gene cluster and compared with the relationships defined based on the *swrA* gene analysis. The same clusters were defined with the *swrA* phylogenetic tree, showing more homogenous clusters (Figure 5).

The genes coding for alanine racemase, carboxymuconolactone decarboxylase, MFS transporter, hypothetical protein, tautomerase, hypothetical protein, hypothetical protein, hypothetical protein, LysR family transcriptional regulator, and ssDNA-binding protein (Figure 5, genes numbered from 60 to 68; Table S5) are exclusive to the *Serratia* sp. YD25^T^ cluster. AntiSMASH cluster prediction identified four additional genes involved in PKS-NRPS, encoding for enoylreductase quinone oxidoreductase and ketoreductase 3-oxoacyl-(acyl-carrier-protein) reductase, both present in all the strains; enoylreductase dehydrogenase, present in seven strains; and aromatic amino acid aminotransferase, present in all strains except in *Serratia* sp. YD25^T^ (Table 5 and Figure 5).

## 4. Discussion

Strains of the genus *Serratia* are known to colonize a diversity of environments. This ability is usually related to the ability to produce lipopeptides, which includes the serrawettins. The physiological roles of such surface-active exolipids are mostly unknown but seem to contribute specifically to enhancing the flagellum-dependent and flagellum-independent spreading growth of the bacteria on a surface environment. Serrawettins were first reported in pigmented *S. marcescens* [4] and 53.6% of *Serratia* strains that showed the *swrW* or *swrA* genes clusters in our study belonged to this species. The analysis of the genomes of 84 *Serratia* strains confirmed that *S. marcescens* is the only species with strains able to produce serrawettin W1 or W2. From the total genomes analyzed, the *swrW* biosynthetic gene clusters were detected in 17 *Serratia* genomes. These strains belonged to different species, namely, eight *S. marcescens*, four *S. plymuthica*, two *S. grimesii*, one *S. nematodiphila*, and two unidentified *Serratia* species. Eleven *Serratia* genomes showed *swrA* biosynthetic gene clusters. The *swrA* biosynthetic gene clusters were detected in strains belonging to the species *S. marcescens* (seven)*, S. ureilytica* (one), and three belong to unidentified *Serratia* species. These strains come from different sources, such as waste water [43], paper machines [34], infected patients [19,44,49,60], pond water, human tissue [35], rapeseed roots [29,30,31], nematodes, buffer solutions [25], different plants [39,61], and soil [27,28].

AntiSMASH software was able to identify the serrawettins gene clusters as NRPS clusters, as previously described [12,13]. When compared with the known serrawettin W1 proteins, the *S. marcescens* strains had a protein with an identity percentage higher than 95%, while proteins of the *S. plymuthica* and *S. grimesii* strains showed an identity higher than 81% and 78%, respectively. From the serrawettin W1 biosynthesis proteins, 70.2% are conserved residues present in all core biosynthetic proteins, suggesting that the serrawettin W1 protein is well conserved among different strains. When compared with the known serrawettin W2 proteins, *S. marcescens* and *S. ureilytica* showed a protein identity higher than 76%, *Serratia* strains YD25 and SCBI higher than 93%, and subspecies of *S. marcescens* higher than 99%. In spite of *swr2* proteins divergence, 62.3% were conserved residues present in all core serrawettin W2 biosynthetic proteins, suggesting a well-conserved serrawettin W2 protein among the different strains.

As previously described by Li et al. [12], all serrawettin W1 biosynthetic genes have a condensation, adenylation, thiolation, and thioesterase domains, also predicted in our study by antiSMASH software. From the serrawettin W1 biosynthetic gene clusters, a total of 80 different genes were identified, 15 common to all clusters. These common genes encode for proteins that may play an important role in serrawettin W1 biosynthesis. AntiSMASH software predicted four PKS-NRPS genes in the serrawettin W1 cluster, which are multi-enzymatic and multi-domain genes involved in the biosynthesis of nonribosomal peptides: an oxidoreductase present in 7 *Serratia* clusters (enoylreductase), a 3-oxoacyl-(acyl-carrier-protein) reductase present in all *Serratia* clusters (ketoreductase), a dehydrogenase present in 14 *Serratia* clusters (enoylreductase), and an aromatic amino acid aminotransferase present in 15 *Serratia* clusters (aminotransferase). Usually, PKS genes consist of the acyltransferase (AT), ketosynthase (KS), and a ketoreductase (KR) domains [13]. Proteins of the serrawettin W1 biosynthetic gene cluster tree and SwrW biosynthesis protein tree, when compared, revealed common clades, suggesting that the *swrW* gene and all cluster organizations have a similar evolutionary history.

Analysis of the *swrA* biosynthetic gene clusters from 11 *Serratia* genomes demonstrated complex serrawettin W2 biosynthesis protein domains with five condensation domains, five adenylation domains, five thiolation domains, and one thioesterase domain, as previously described in Su et al. [13]. From the *swrA* biosynthetic gene clusters, a total of 68 different proteins were identified, 24 common to all clusters, and 8 exclusive to the *Serratia* sp. YD25 biosynthetic gene cluster. These common proteins may play an important role in serrawettin W2 biosynthesis and may be involved in the different steps needed to produce a nonribosomal peptide as DNA binding, adenylation, condensation, thiolation, and thioesterase. According to Su et al. [13], the three proteins identified as involved in PKS-NRPS hybrid polyketide synthase (acyltransferase, ketosynthase, and ketoreductase) are also involved in the serrawettin W2 biosynthesis process. Our results of the serrawettin W2 biosynthetic gene clusters revealed four genes identified as part of the PKS-NRPS system, such as oxireductase and 3-oxoacyl-(acyl-carrier-protein) reductase, both present in all serrawettin W2 biosynthesis protein clusters; a dehydrogenase present in seven clusters; and an aromatic amino acid aminotransferase present in nine clusters. Proteins of the serrawettin W2 gene clusters tree and SwrA biosynthesis protein tree, when compared, revealed common groups in both trees, suggesting that not only the serrawettin W2 biosynthesis gene but also all cluster organizations have a similar evolutionary history.

None of the genomes analyzed included both the serrawettin biosynthetic gene clusters. Although both present in the *Serratia* genus, the two biosynthetic gene clusters are distributed in the two clades, as revealed in the 16S rRNA gene-defined phylogenetic tree of the genus *Serratia*. W1 is present in two clades, in five *S. marcescens*, one *S. nematodiphila*, and in all the strains of the genus *S. plymuthica* analyzed, except one, and in two *S. grimesii* and one *S. rubidaea*. On the other hand, W2 is common in all strains of the *S. marcescens* sub-cluster and three additional strains of the same species, but it was not present in the other *Serratia* clade.

This work identified four genes common to all serrawettin gene clusters, highlighting their essential potential in the serrawettins biosynthetic process. These genes encoding for CTP synthase, glyoxalase/bleomycin resistance protein/dioxygenase, LrgA family protein, and LrgB family protein are flanking the biosynthesis genes *swrW* and *swrA*. In both organizations, the genes encoding for CTP synthase is immediately upstream of the serrawettin biosynthetic gene and the ones encoding for the LrgA and LrgB family proteins are immediately downstream of the genes. Glyoxalase/bleomycin resistance protein/dioxygenase is downstream of *swrW* and *swrA*, with a group of non-conserved genes between them. The *lrgAB* operon in the *Staphylococcus* codes for a transmembrane protein. The LrgA protein shares many characteristics with bacteriophage antiholins [62]. The antiholin homologue in *Bacillus subtilis* transports pyruvate and it is regulated in an unconventional way by its substrate molecule [63]. Holins and antiholins control the formation of channels for murein hydrolase to export across the bacterial membrane to time the bacteriophage-induced cell lysis [64]. In *Serratia,* this operon seems to be associated with the transport of serrawettin, as a facilitator, independently of the coding gene (*swrW* or *swrA*), and, therefore, of the complexity of the molecule. The holing-antiholin class of proteins was originally discovered in bacteriophages, where they modulate host cell lysis during lytic infection [65]. A hypothetical model suggests that these proteins could have been acquired by horizontal gene transfer to an ancient bacterium through the integration of these elements into its genome [66]. This suggests the introduction of *swrW* and *swrA* as two independent events in the *lrgAB* operon, but more work is needed to understand the evolution and functional diversification of serrawettin.

Within the W1 biosynthetic gene cluster, the gene coding for a quinone oxidoreductase YhdH/YhfP family is present only in two strains of *S. marcescens* and in three strains from different species of *Serratia*. These *Serratia* strains are missing the gene cluster characterized by the Major Facilitator Superfamily (MFS) gene present in all the other *S. marcescens* strains. MFS is one of the two largest families of membrane transporters found in bacteria [67]. Phylogenetic analyses revealed the occurrence of 17 distinct families within the MFS, each of which generally transports a single class of compounds. This suggests that within the W1 cluster, the MFS transport system is conserved for most *S. marcescens* although the other *Serratia* species presented the quinone oxidoreductase system YhdH. 

The W2 biosynthetic gene cluster has a more conserved genetic organization and 24 genes were common to all strains. The gene clusters composed of cystine ABC transporter, substrate-binding protein, and the alanine racemase seem to be involved in the selective transport of amino acids into the cell and in the alanine L to D interconversion. These systems may facilitate the amino acid acquisition by the cell for W2 synthesis. In the W2 biosynthetic gene cluster, both genes are present in several *Serratia* species but only in four strains of *S. marcescens*. Both the mechanistic studies, kinetic and energetic, are needed to relate the genes’ presence with W2 synthesis in these strains. The other four strains producing the serrawettin W2, which do not present the gene cluster with ABC transporter and racemase, show a gene cluster including a gene belonging to the cyclase family protein. In *Serratia*, the cyclase family protein shows high homology with diguanylate cyclase, showing a domain from the GGDEF family protein [68]. They are used as an intracellular signaling molecule regulator, involved in bacterial biofilm formation, and persist in several bacteria species.

## 5. Conclusions

In conclusion, the present work shows that most species of the genus *Serratia* that already have their genome sequenced have clusters of serrawettin biosynthetic genes in their genomes. AntiSMASH software was able to identify the serrawettins gene clusters as NRPS clusters. The grouping of biosynthetic gene clusters W1 and W2 are mutually exclusive in the genome. Moreover, the *swrW* and *swrA* biosynthetic genes are framed by the same genes in the biosynthetic clusters. CTP is upstream and the operon *LgrAB* is downstream, suggesting a horizontal gene acquisition of the biosynthetic system by an ancient strain. Within the W1 biosynthetic cluster, the genes coding for the quinone oxidoreductase YhdH/YhfP family, and the one coding for the Major Facilitator Superfamily, are mutually exclusive in the genomes of the strains. The same is found in the W2 biosynthetic gene cluster, in the genes cystine ABC transporter, substrate-binding protein, and the alanine racemase and cyclase family proteins are also mutually exclusive.

## Figures and Tables

**Table 1:** *Serratia* strains with the *swrW* and *swrA* genes identified through antiSMASH software. AntiSMASH software was used in 84 *Serratia* genomes (see Table S1). X represents the presence of genes *swrW* and *swrA* in seventeen and eleven genomes, respectively.

Bacterial Strain	Accession Number	*swrW*	*swrA*
*Serratia* sp. AS12	CP002774.1	X	
*Serratia* sp. AS13	CP002775.1	X	
*Serratia* sp. FS14	CP005927.1	X	
*Serratia* sp. SCBI	CP003424.1		X
*Serratia* sp. YD25	CP016948.1		X
*Serratia* sp. SSNIH1	CP026383.1		X
*Serratia* sp. PWN146	LT575490.1		X
*Serratia marcescens* strain UMH8	CP018927.1	X	
*Serratia marcescens* strain IOMTU 115	AB894481.1	X	
*Serratia marcescens subsp. marcescens* ATCC 13880	JMPQ01000033.1	X	
*Serratia marcescens* strain CDC_813-60 DP21	JOVM01000004.1	X	
*Serratia nematodiphila* DZ0503SBS1 strain DSM 21420	JPUX00000000.1	X	
*Serratia marcescens* VGH107	AORJ00000000.1	X	
*Serratia marcescens* EGD-HP20	AVSR00000000.1	X	
*Serratia marcescens* WW4	CP003959.1	X	
*Serratia marcescens* BIDMC 81	JJMZ01000006.1	X	
*Serratia* strain TEL NODE_13	LDEG01000018.1	X	
*Serratia plymuthica* NBRC 102599^T^	BCTU00000000.1	X	
*Serratia grimesii* isolate BXF1	LT883155.1	X	
*Serratia grimesii* strain A2	JGVP00000000.1	X	
*Serratia plymuthica* AS9	CP002773.1	X	
*Serratia marcescens* SM39	AP013063.1		X
*Serratia marcescens* SmUNAM836	CP012685.1		X
*Serratia marcescens* BIDMC 44	JAPD01000005.1		X
*Serratia ureilytica* Lr5/4 LG59	JSFB01000001.1		X
*Serratia marcescens* RSC-14	CP012639.1		X
*Serratia marcescens* subsp. *marcescens* AH0650_Sm1 AG2	LFJS01000014.1		X
*Serratia marcescens* subsp. *marcescens* Db11	HG326223.1		X

**Table 2:** The serrawettin W1 gene (*swrW*) of each strain in this study and the accession numbers and identification of the *swrW* gene’s closest relatives using BLASTP.

Bacterial Strain	*swrW* Closest Relative Genes	Accession Number	Identity Percentage
*Serratia marcescens* EGD-HP20	non-ribosomal peptide synthetase	ERH70695.1	99.52
*Serratia marcescens* WW4	serrawettin W1 synthetase	AGE20181.1	100
*Serratia* sp. FS14	putative serrawettin W1 synthetase	AIA46701.1	100
*Serratia marcescens* VGH107	amino acid adenylation protein	EMF04443.1	99.12
*Serratia nematodiphila* DZ0503SBS1 strain DSM 21420	non-ribosomal peptide synthetase	KFF87803.1	99.31
*Serratia marcescens* strain IOMTU 115	putative serrawettin W1 synthetase	BAO21138.1	99.92
*Serratia marcescens* strain UMH8	non-ribosomal peptide synthetase	ASM18665.1	99.01
*Serratia marcescens* subsp. *marcescens* ATCC 13880	amino acid adenylation domain-containing protein	KFD14984.1	98.57
*Serratia marcescens* strain CDC_813-60 DP21	non-ribosomal peptide synthetase	KFL05097.1	98.63
*Serratia marcescens* BIDMC 81	non-ribosomal peptide synthetase	EZQ62923.1	95.12
*Serratia* strain TEL NODE_13	non-ribosomal peptide synthetase	KLE36484.1	95.05
*Serratia plymuthica* NBRC 102599T	non-ribosomal peptide synthetase	WP_063202307.1	81.71
*Serratia* sp. AS13	non-ribosomal peptide synthetase	AEG30284.1	81.48
*Serratia plymuthica* AS9	non-ribosomal peptide synthetase	AEF47625.1	81.48
*Serratia* sp. AS12	non-ribosomal peptide synthetase	WP_013814722.1	81.48
*Serratia grimesii* isolate BXF1	amino acid adenylation domain-containing protein	SMZ58711.1	77.79
*Serratia grimesii* strain A2	non-ribosomal peptide synthetase	KFB89923.1	78.17

**Table 3:** The PKS gene accession numbers from the *swrW* biosynthetic gene clusters predicted by antiSMASH software.

Bacterial Strain	Enoylreductase Quinone Oxidoreductase	Ketoreductase 3-Oxoacyl-(Acyl-Carrier-Protein) Reductase	Enoylreductase Dehydrogenase	Aromatic Amino Acid Aminotransferase
*Serratia marcescens* EGD-HP20		ERH70706.1	ERH70710.1	ERH70714.1
*Serratia marcescens* WW4		AGE20192.1	AGE20197.1	AGE20201.1
*Serratia* sp. FS14		AIA46690.1	AIA46685.1	AIA46681.1
*Serratia marcescens* VGH107		EMF04432.1	EMF04427.1	EMF04423.1
*Serratia nematodiphila* DZ0503SBS1 strain DSM 21420		KFF87792.1	KFF87787.1	KFF87783.1
*Serratia marcescens* strain IOMTU 115		BAO21148.1	BAO21153.1	BAO21155.1
*Serratia marcescens* strain UMH8		ASM18675.1	ASM18680.1	ASM18684.1
*Serratia marcescens subsp. Marcescens* ATCC 13880		KFD14974.1	KFD14969.1	KFD14965.1
*Serratia marcescens* strain CDC_813-60 DP21		KFL04091.1	KFL03204.1	KFL04717.1
*Serratia marcescens* BIDMC 81	EZQ62938.1	EZQ62913.1		EZQ62903.1
*Serratia* strain TEL NODE_13	KLE36470.1	KLE36494.1		KLE36503.1
*Serratia plymuthica* NBRC 102599^T^		WP_063202297.1	WP_062868864.1	WP_006328339.1
*Serratia* sp. AS13	AEG30270.1	AEG30294.1	AEG30297.1	AEG30301.1
*Serratia plymuthica* AS9	AEF47611.1	AEF47635.1	AEF47638.1	AEF47642.1
*Serratia* sp. AS12	WP_013814712.1	WP_013814732.1	WP_013814734.1	WP_013814736.1
*Serratia grimesii* isolate BXF1	SMZ58698.1	SMZ58721.1	SMZ58727.1	SMZ58731.1
*Serratia grimesii* strain A2	KFB89936.1	KFB89913.1		KFB89904.1

**Table 4:** The serrawettin W2 gene (*swrA*) of each strain in this study and the accession numbers and identification of the *swrA* gene’s closest relatives using BLASTX.

Bacterial Strain	*swrA* Closest Relative Genes	Accession Number	Identity Percentage
*Serratia marcescens* SM39	*Serratia marcescens* SM39 DNA, complete genome	BAO35825.1	76.70
*Serratia marcescens* SmUNAM836	*Serratia marcescens* strain SmUNAM836, complete genome	ALE98111.1	76.70
*Serratia* sp. SSNIH1	*Serratia* sp. SSNIH1 chromosome, complete genome	AUY16873.1	76.70
*Serratia* sp. PWN146	*Serratia marcescens* isolate PWN146_assembly genome assembly, chromosome	SAY45247.1	77.02
*Serratia ureilytica* Lr5/4 LG59	*Serratia marcescens* strain UMH7, complete genome	KKO55915.1	76.38
*Serratia marcescens* RSC-14	*Serratia marcescens* strain RSC-14, complete genome	ALD45109.1	76.38
*Serratia marcescens* BIDMC 44	*Serratia marcescens* strain 1274 genome	ETX44761.1	77.35
*Serratia* sp. YD25	*Serratia* sp. YD25, complete genome	AOF02338.1	93.57
*Serratia* sp. SCBI	*Serratia* sp. SCBI, complete genome	AIM23801.1	93.44
*Serratia marcescens* subsp. *marcescens* AH0650_Sm1 AG2	*Serratia marcescens* strain BWH-23 chromosome, complete genome	KMU50701.1	99.40
*Serratia marcescens* subsp. *marcescens* Db11	*Serratia marcescens* subsp. *marcescens* Db11, complete genome	CDG14244.1	99.32

**Table 5:** The PKS genes from the swrA biosynthetic gene clusters predicted by antiSMASH software.

Bacterial Strain	Enoylreductase Quinone Oxidoreductase	Ketoreductase 3-Oxoacyl-(Acyl-Carrier-Protein) Reductase	Enoylreductase Dehydrogenase	Aminotransferase
*Serratia marcescens* SM39	BAO35810.1	BAO35835.1	BAO35838.1	BAO35842.1
*Serratia marcescens* SmUNAM836	ALE98097.1	ALE98121.1	ALE98124.1	ALE98128.1
*Serratia* sp. SSNIH1	AUY16858.1	AUY16883.1	AUY16886.1	AUY16890.1
*Serratia* sp. PWN146	SAY45233.1	SAY45257.1	SAY45263.1	SAY45267.1
*Serratia ureilytica* Lr5/4 LG59	KKO55998.1	KKO58381.1		KKO57271.1
*Serratia marcescens* RSC-14	ALD45123.1	ALD45099.1		ALD45091.1
*Serratia marcescens* BIDMC 44	ETX44746.1	ETX44771.1	ETX44774.1	ETX44778.1
*Serratia* sp. YD25	AOF01119.1	AOF01143.1		
*Serratia* sp. SCBI	AIM23787.1	AIM23811.1		AIM23821.1
*Serratia marcescens* subsp. *marcescens* AH0650_Sm1 AG2	KMU50686.1	KMU50711.1	KMU50716.1	KMU50720.1
*Serratia marcescens* subsp. *marcescens* Db11	CDG14228.1	CDG14254.1	CDG14259.1	CDG14263.1

## Supplementary Materials

The following are available online at https://www.mdpi.com/2079-7737/9/12/482/s1, Table S1. General information of *Serratia* strains used in this study. Table S2. Serrawettin W1 gene (*swrW*) of each strain predicted by antiSMASH in *Serratia* strains ATCC 13880, CDC_813-60 DP21, UMH8, IOMTU 115, DSM 21420, VGH107, EGD-HP20, WW4, FS14, BIDMC81, TEL NODE_13, NBRC 102599, BXF1, A2, AS13, AS9 and AS12, in this study, and the accession numbers and identification of *swrW* closest relatives using BLASTP. Table S3. Serrawettin W2 gene (*swrA*) of each strain predicted by antiSMASH in *Serratia* strains PWN146, SSNIH1, SM39, SmUNAm836, BIDMC44, Lr5/4 LG59, RSC-14, AH0650, Db11, SCBI and YD25, in this study, and the accession numbers and identification of *swrA* closest relatives using BLASTX. Table S4. Accession numbers of identified proteins of serrawettin W1 biosynthetic gene clusters of *Serratia* strains ATCC 13880, CDC_813-60 DP21, UMH8, IOMTU 115, DSM 21420, VGH107, EGD-HP20, WW4, FS14, BIDMC81, TEL NODE_13, NBRC 102599, BXF1, A2, AS13, AS9 and AS12 by antiSMASH and NCBI BLASTP software. Table S5. Accession numbers of identified proteins of serrawettin W2 biosynthetic gene clusters of *Serratia* strains PWN146, SSNIH1, SM39, SmUNAm836, BIDMC44, Lr5/4 LG59, RSC-14, AH0650, Db11, SCBI and YD25 by antiSMASH and NCBI BLASTX software.
